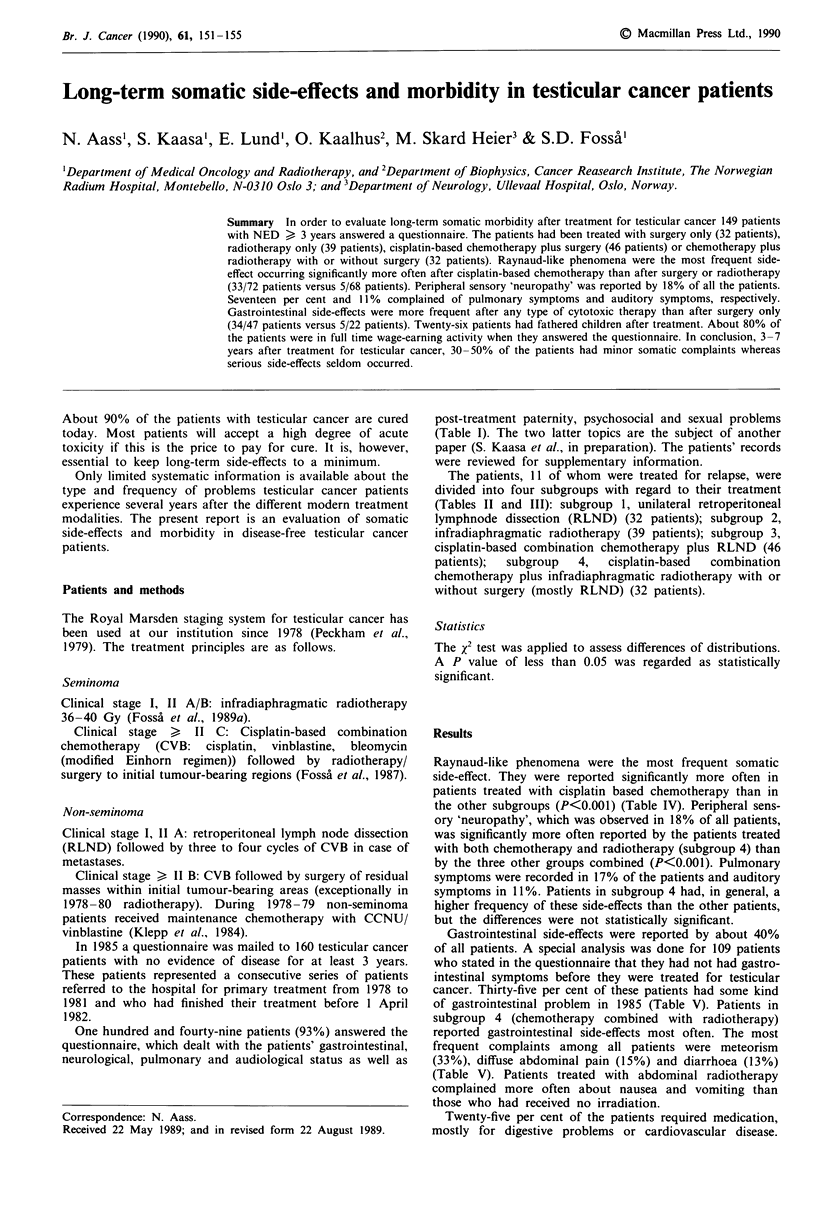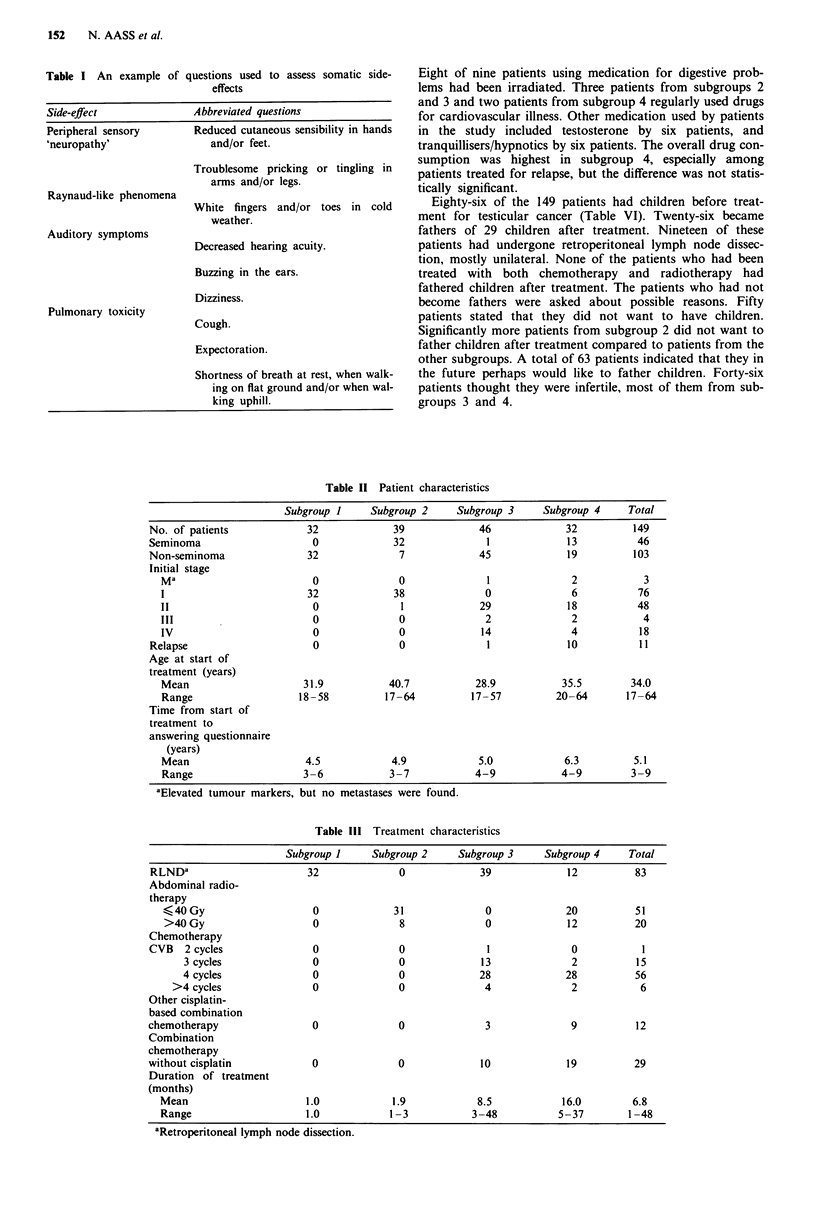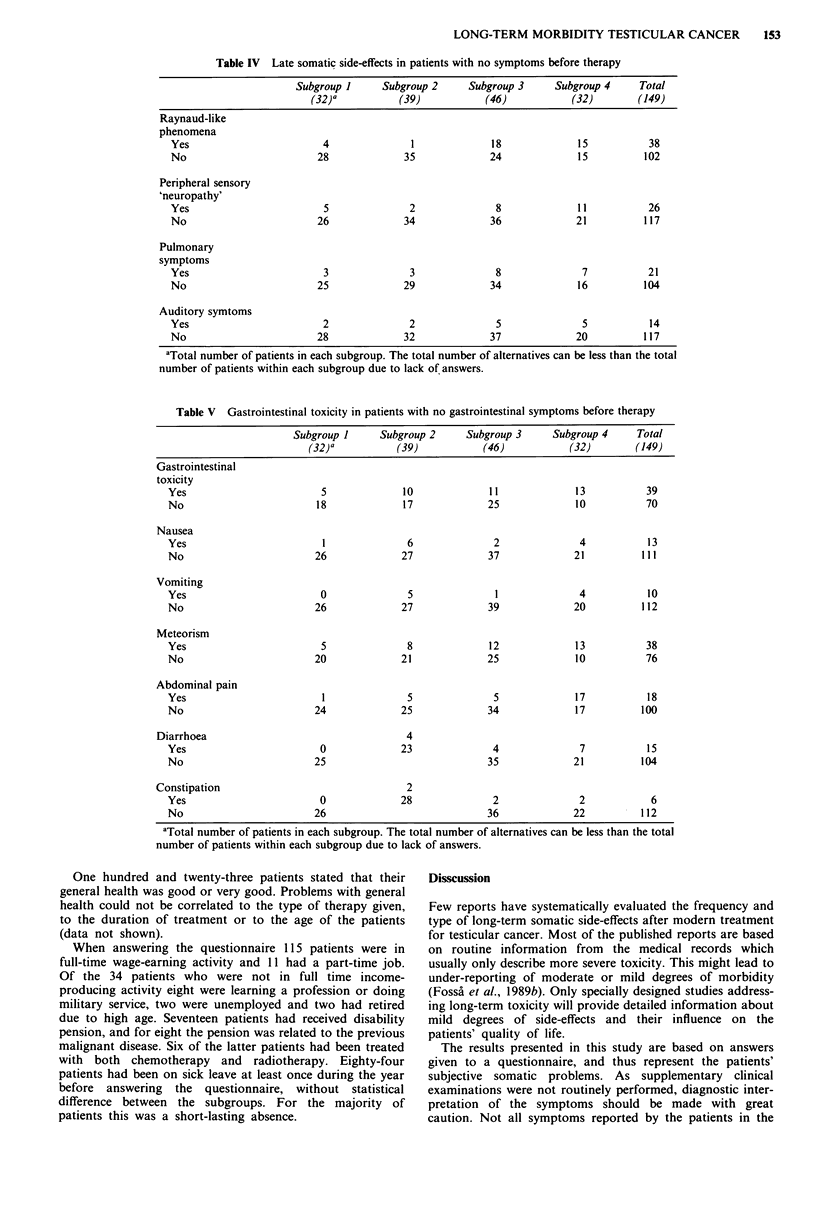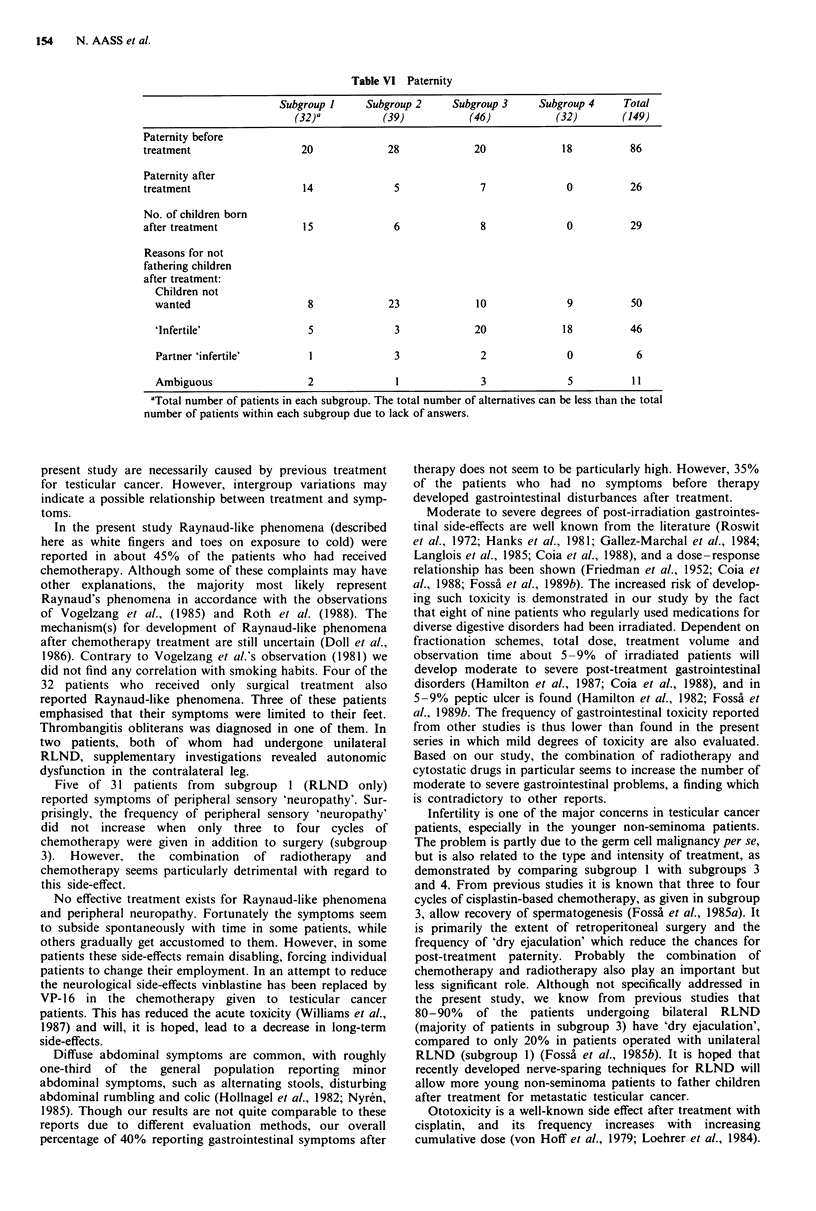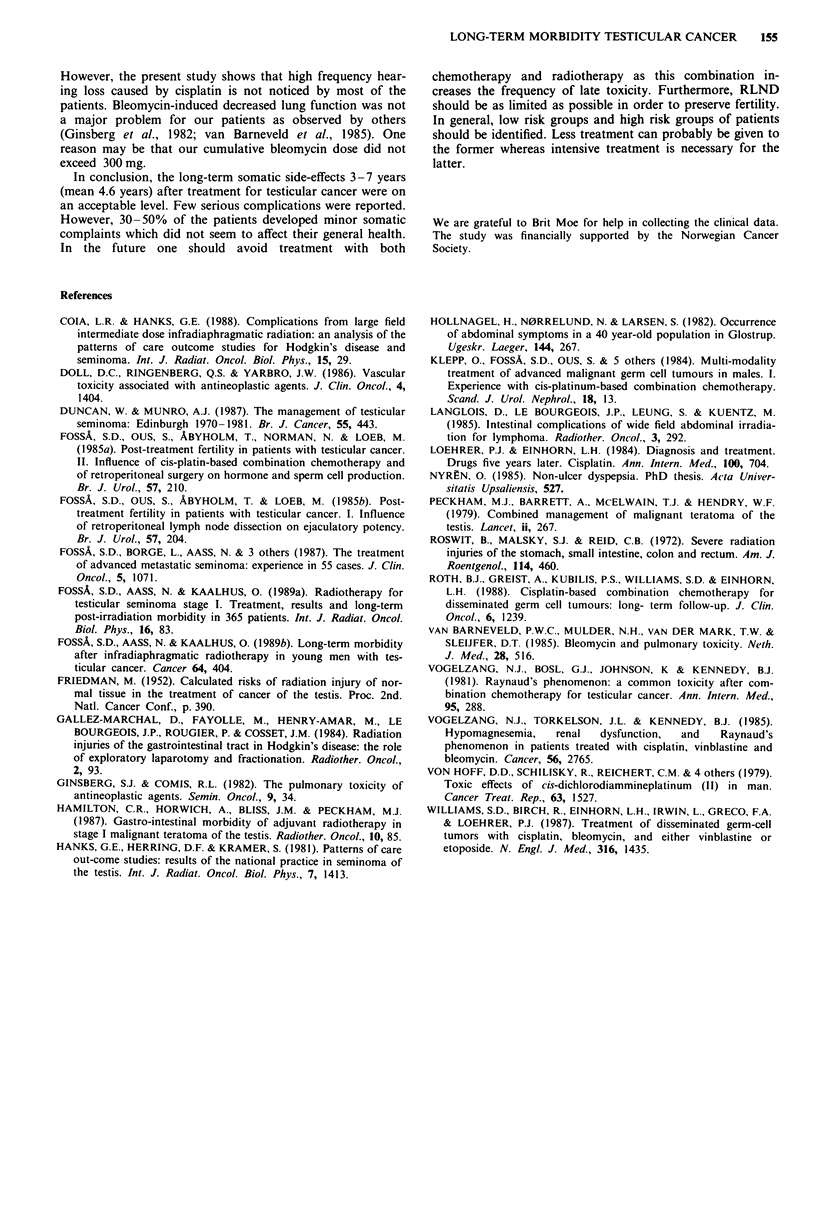# Long-term somatic side-effects and morbidity in testicular cancer patients.

**DOI:** 10.1038/bjc.1990.31

**Published:** 1990-01

**Authors:** N. Aass, S. Kaasa, E. Lund, O. Kaalhus, M. S. Heier, S. D. Fosså

**Affiliations:** Department of Medical Oncology and Radiotherapy, Norwegian Radium Hospital, Montebello, Oslo.

## Abstract

In order to evaluate long-term somatic morbidity after treatment for testicular cancer 149 patients with NED greater than or equal to 3 years answered a questionnaire. The patients had been treated with surgery only (32 patients, radiotherapy only (39 patients), cisplatin-based chemotherapy plus surgery (46 patients) or chemotherapy plus radiotherapy with or without surgery (32 patients). Raynaud-like phenomena were the most frequent side-effect occurring significantly more often after cisplatin-based chemotherapy than after surgery or radiotherapy (33/72 patients versus 5/68 patients). Peripheral sensory 'neuropathy' was reported by 18% of all the patients. Seventeen per cent and 11% complained of pulmonary symptoms and auditory symptoms, respectively. Gastrointestinal side-effects were more frequent after any type of cytotoxic therapy than after surgery only (34/47 patients versus 5/22 patients). Twenty-six patients had fathered children after treatment. About 80% of the patients were in full time wage-earning activity when they answered the questionnaire. In conclusion, 3-7 years after treatment for testicular cancer, 30-50% of the patients had minor somatic complaints whereas serious side-effects seldom occurred.


*Br. J. Cancer* (1990), **61**, 151–155 © Macmillan Press Ltd., 1990

### Long-term somatic side-effects and morbidity in testicular cancer patients

N. Aass[1], S. Kaasa[1], E. Lund[1], O. Kaalhus[2], M. Skard Heier[3] & S.D. Fosså[1]

[1]*Department of Medical Oncology and Radiotherapy, and* [2]*Department of Biophysics, Cancer Reasearch Institute, The Norwegian Radium Hospital, Montebello, N-0310 Oslo 3; and* [3]*Department of Neurology, Ullevaal Hospital, Oslo, Norway.*

**Summary** In order to evaluate long-term somatic morbidity after treatment for testicular cancer 149 patients with NED ⩾ 3 years answered a questionnaire. The patients had been treated with surgery only (32 patients), radiotherapy only (39 patients), cisplatin-based chemotherapy plus surgery (46 patients) or chemotherapy plus radiotherapy with or without surgery (32 patients). Raynaud-like phenomena were the most frequent side-effect occurring significantly more often after cisplatin-based chemotherapy than after surgery or radiotherapy (33/72 patients versus 5/68 patients). Peripheral sensory 'neuropathy' was reported by 18% of all the patients. Seventeen per cent and 11% complained of pulmonary symptoms and auditory symptoms, respectively. Gastrointestinal side-effects were more frequent after any type of cytotoxic therapy than after surgery only (34/47 patients versus 5/22 patients). Twenty-six patients had fathered children after treatment. About 80% of the patients were in full time wage-earning activity when they answered the questionnaire. In conclusion, 3–7 years after treatment for testicular cancer, 30–50% of the patients had minor somatic complaints whereas serious side-effects seldom occurred.

About 90% of the patients with testicular cancer are cured today. Most patients will accept a high degree of acute toxicity if this is the price to pay for cure. It is, however, essential to keep long-term side-effects to a minimum.

Only limited systematic information is available about the type and frequency of problems testicular cancer patients experience several years after the different modern treatment modalities. The present report is an evaluation of somatic side-effects and morbidity in disease-free testicular cancer patients.

### Patients and methods

The Royal Marsden staging system for testicular cancer has been used at our institution since 1978 (Peckham *et al.*, 1979). The treatment principles are as follows.

### *Seminoma*

Clinical stage I, II A/B: infradiaphragmatic radiotherapy 36–40 Gy (Fosså *et al.*, 1989*a*).

Clinical stage ⩾ II C: Cisplatin-based combination chemotherapy (CVB: cisplatin, vinblastine, bleomycin (modified Einhorn regimen)) followed by radiotherapy/surgery to initial tumour-bearing regions (Fosså *et al.*, 1987).

### *Non-seminoma*

Clinical stage I, II A: retroperitoneal lymph node dissection (RLND) followed by three to four cycles of CVB in case of metastases.

Clinical stage ⩾ II B: CVB followed by surgery of residual masses within initial tumour-bearing areas (exceptionally in 1978–80 radiotherapy). During 1978–79 non-seminoma patients received maintenance chemotherapy with CCNU/vinblastine (Klepp *et al.*, 1984).

In 1985 a questionnaire was mailed to 160 testicular cancer patients with no evidence of disease for at least 3 years. These patients represented a consecutive series of patients referred to the hospital for primary treatment from 1978 to 1981 and who had finished their treatment before 1 April 1982.

One hundred and fourty-nine patients (93%) answered the questionnaire, which dealt with the patients' gastrointestinal, neurological, pulmonary and audiological status as well as post-treatment paternity, psychosocial and sexual problems (Table I). The two latter topics are the subject of another paper (S. Kaasa *et al.*, in preparation). The patients' records were reviewed for supplementary information.

The patients, 11 of whom were treated for relapse, were divided into four subgroups with regard to their treatment (Tables II and III): subgroup 1, unilateral retroperitoneal lymphnode dissection (RLND) (32 patients); subgroup 2, infradiaphragmatic radiotherapy (39 patients); subgroup 3, cisplatin-based combination chemotherapy plus RLND (46 patients); subgroup 4, cisplatin-based combination chemotherapy plus infradiaphragmatic radiotherapy with or without surgery (mostly RLND) (32 patients).

### *Statistics*

The $\chi^2$ test was applied to assess differences of distributions. A $P$ value of less than 0.05 was regarded as statistically significant.

### Results

Raynaud-like phenomena were the most frequent somatic side-effect. They were reported significantly more often in patients treated with cisplatin based chemotherapy than in the other subgroups ($P<0.001$) (Table IV). Peripheral sensory 'neuropathy', which was observed in 18% of all patients, was significantly more often reported by the patients treated with both chemotherapy and radiotherapy (subgroup 4) than by the three other groups combined ($P<0.001$). Pulmonary symptoms were recorded in 17% of the patients and auditory symptoms in 11%. Patients in subgroup 4 had, in general, a higher frequency of these side-effects than the other patients, but the differences were not statistically significant.

Gastrointestinal side-effects were reported by about 40% of all patients. A special analysis was done for 109 patients who stated in the questionnaire that they had not had gastro-intestinal symptoms before they were treated for testicular cancer. Thirty-five per cent of these patients had some kind of gastrointestinal problem in 1985 (Table V). Patients in subgroup 4 (chemotherapy combined with radiotherapy) reported gastrointestinal side-effects most often. The most frequent complaints among all patients were meteorism (33%), diffuse abdominal pain (15%) and diarrhoea (13%) (Table V). Patients treated with abdominal radiotherapy complained more often about nausea and vomiting than those who had received no irradiation.

Twenty-five per cent of the patients required medication, mostly for digestive problems or cardiovascular disease.

Correspondence: N. Aass.
Received 22 May 1989; and in revised form 22 August 1989.

**Table I** An example of questions used to assess somatic side-effects

| *Side-effect* | *Abbreviated questions* |
|---|---|
| Peripheral sensory 'neuropathy' | Reduced cutaneous sensibility in hands and/or feet. |
| | Troublesome pricking or tingling in arms and/or legs. |
| Raynaud-like phenomena | White fingers and/or toes in cold weather. |
| Auditory symptoms | Decreased hearing acuity. |
| | Buzzing in the ears. |
| | Dizziness. |
| Pulmonary toxicity | Cough. |
| | Expectoration. |
| | Shortness of breath at rest, when walking on flat ground and/or when walking uphill. |

Eight of nine patients using medication for digestive problems had been irradiated. Three patients from subgroups 2 and 3 and two patients from subgroup 4 regularly used drugs for cardiovascular illness. Other medication used by patients in the study included testosterone by six patients, and tranquillisers/hypnotics by six patients. The overall drug consumption was highest in subgroup 4, especially among patients treated for relapse, but the difference was not statistically significant.

Eighty-six of the 149 patients had children before treatment for testicular cancer (Table VI). Twenty-six became fathers of 29 children after treatment. Nineteen of these patients had undergone retroperitoneal lymph node dissection, mostly unilateral. None of the patients who had been treated with both chemotherapy and radiotherapy had fathered children after treatment. The patients who had not become fathers were asked about possible reasons. Fifty patients stated that they did not want to have children. Significantly more patients from subgroup 2 did not want to father children after treatment compared to patients from the other subgroups. A total of 63 patients indicated that they in the future perhaps would like to father children. Forty-six patients thought they were infertile, most of them from subgroups 3 and 4.

**Table II** Patient characteristics

| | *Subgroup 1* | *Subgroup 2* | *Subgroup 3* | *Subgroup 4* | *Total* |
|---|---|---|---|---|---|
| No. of patients | 32 | 39 | 46 | 32 | 149 |
| Seminoma | 0 | 32 | 1 | 13 | 46 |
| Non-seminoma | 32 | 7 | 45 | 19 | 103 |
| Initial stage | | | | | |
| M[a] | 0 | 0 | 1 | 2 | 3 |
| I | 32 | 38 | 0 | 6 | 76 |
| II | 0 | 1 | 29 | 18 | 48 |
| III | 0 | 0 | 2 | 2 | 4 |
| IV | 0 | 0 | 14 | 4 | 18 |
| Relapse | 0 | 0 | 1 | 10 | 11 |
| Age at start of treatment (years) | | | | | |
| Mean | 31.9 | 40.7 | 28.9 | 35.5 | 34.0 |
| Range | 18-58 | 17-64 | 17-57 | 20-64 | 17-64 |
| Time from start of treatment to answering questionnaire (years) | | | | | |
| Mean | 4.5 | 4.9 | 5.0 | 6.3 | 5.1 |
| Range | 3-6 | 3-7 | 4-9 | 4-9 | 3-9 |

[a]Elevated tumour markers, but no metastases were found.

**Table III** Treatment characteristics

| | *Subgroup 1* | *Subgroup 2* | *Subgroup 3* | *Subgroup 4* | *Total* |
|---|---|---|---|---|---|
| RLND[a] | 32 | 0 | 39 | 12 | 83 |
| Abdominal radiotherapy | | | | | |
| $\leqslant$40 Gy | 0 | 31 | 0 | 20 | 51 |
| >40 Gy | 0 | 8 | 0 | 12 | 20 |
| Chemotherapy | | | | | |
| CVB 2 cycles | 0 | 0 | 1 | 0 | 1 |
| 3 cycles | 0 | 0 | 13 | 2 | 15 |
| 4 cycles | 0 | 0 | 28 | 28 | 56 |
| >4 cycles | 0 | 0 | 4 | 2 | 6 |
| Other cisplatin-based combination chemotherapy | 0 | 0 | 3 | 9 | 12 |
| Combination chemotherapy without cisplatin | 0 | 0 | 10 | 19 | 29 |
| Duration of treatment (months) | | | | | |
| Mean | 1.0 | 1.9 | 8.5 | 16.0 | 6.8 |
| Range | 1.0 | 1-3 | 3-48 | 5-37 | 1-48 |

[a]Retroperitoneal lymph node dissection.

**Table IV** Late somatic side-effects in patients with no symptoms before therapy

| | *Subgroup 1 (32)*[a] | *Subgroup 2 (39)* | *Subgroup 3 (46)* | *Subgroup 4 (32)* | *Total (149)* |
|---|---|---|---|---|---|
| Raynaud-like phenomena | | | | | |
| Yes | 4 | 1 | 18 | 15 | 38 |
| No | 28 | 35 | 24 | 15 | 102 |
| Peripheral sensory 'neuropathy' | | | | | |
| Yes | 5 | 2 | 8 | 11 | 26 |
| No | 26 | 34 | 36 | 21 | 117 |
| Pulmonary symptoms | | | | | |
| Yes | 3 | 3 | 8 | 7 | 21 |
| No | 25 | 29 | 34 | 16 | 104 |
| Auditory symtoms | | | | | |
| Yes | 2 | 2 | 5 | 5 | 14 |
| No | 28 | 32 | 37 | 20 | 117 |

[a]Total number of patients in each subgroup. The total number of alternatives can be less than the total number of patients within each subgroup due to lack of answers.

**Table V** Gastrointestinal toxicity in patients with no gastrointestinal symptoms before therapy

| | *Subgroup 1 (32)*[a] | *Subgroup 2 (39)* | *Subgroup 3 (46)* | *Subgroup 4 (32)* | *Total (149)* |
|---|---|---|---|---|---|
| Gastrointestinal toxicity | | | | | |
| Yes | 5 | 10 | 11 | 13 | 39 |
| No | 18 | 17 | 25 | 10 | 70 |
| Nausea | | | | | |
| Yes | 1 | 6 | 2 | 4 | 13 |
| No | 26 | 27 | 37 | 21 | 111 |
| Vomiting | | | | | |
| Yes | 0 | 5 | 1 | 4 | 10 |
| No | 26 | 27 | 39 | 20 | 112 |
| Meteorism | | | | | |
| Yes | 5 | 8 | 12 | 13 | 38 |
| No | 20 | 21 | 25 | 10 | 76 |
| Abdominal pain | | | | | |
| Yes | 1 | 5 | 5 | 17 | 18 |
| No | 24 | 25 | 34 | 17 | 100 |
| Diarrhoea | | 4 | | | |
| Yes | 0 | 23 | 4 | 7 | 15 |
| No | 25 | | 35 | 21 | 104 |
| Constipation | | 2 | | | |
| Yes | 0 | 28 | 2 | 2 | 6 |
| No | 26 | | 36 | 22 | 112 |

[a]Total number of patients in each subgroup. The total number of alternatives can be less than the total number of patients within each subgroup due to lack of answers.

One hundred and twenty-three patients stated that their general health was good or very good. Problems with general health could not be correlated to the type of therapy given, to the duration of treatment or to the age of the patients (data not shown).

When answering the questionnaire 115 patients were in full-time wage-earning activity and 11 had a part-time job. Of the 34 patients who were not in full time income-producing activity eight were learning a profession or doing military service, two were unemployed and two had retired due to high age. Seventeen patients had received disability pension, and for eight the pension was related to the previous malignant disease. Six of the latter patients had been treated with both chemotherapy and radiotherapy. Eighty-four patients had been on sick leave at least once during the year before answering the questionnaire, without statistical difference between the subgroups. For the majority of patients this was a short-lasting absence.

### Disscussion

Few reports have systematically evaluated the frequency and type of long-term somatic side-effects after modern treatment for testicular cancer. Most of the published reports are based on routine information from the medical records which usually only describe more severe toxicity. This might lead to under-reporting of moderate or mild degrees of morbidity (Fosså *et al.*, 1989*b*). Only specially designed studies addressing long-term toxicity will provide detailed information about mild degrees of side-effects and their influence on the patients' quality of life.

The results presented in this study are based on answers given to a questionnaire, and thus represent the patients' subjective somatic problems. As supplementary clinical examinations were not routinely performed, diagnostic interpretation of the symptoms should be made with great caution. Not all symptoms reported by the patients in the

**Table VI** Paternity

| | *Subgroup 1 (32)*[a] | *Subgroup 2 (39)* | *Subgroup 3 (46)* | *Subgroup 4 (32)* | *Total (149)* |
|---|---|---|---|---|---|
| Paternity before treatment | 20 | 28 | 20 | 18 | 86 |
| Paternity after treatment | 14 | 5 | 7 | 0 | 26 |
| No. of children born after treatment | 15 | 6 | 8 | 0 | 29 |
| Reasons for not fathering children after treatment: | | | | | |
| Children not wanted | 8 | 23 | 10 | 9 | 50 |
| 'Infertile' | 5 | 3 | 20 | 18 | 46 |
| Partner 'infertile' | 1 | 3 | 2 | 0 | 6 |
| Ambiguous | 2 | 1 | 3 | 5 | 11 |

[a]Total number of patients in each subgroup. The total number of alternatives can be less than the total number of patients within each subgroup due to lack of answers.

present study are necessarily caused by previous treatment for testicular cancer. However, intergroup variations may indicate a possible relationship between treatment and symptoms.

In the present study Raynaud-like phenomena (described here as white fingers and toes on exposure to cold) were reported in about 45% of the patients who had received chemotherapy. Although some of these complaints may have other explanations, the majority most likely represent Raynaud's phenomena in accordance with the observations of Vogelzang *et al.*, (1985) and Roth *et al.* (1988). The mechanism(s) for development of Raynaud-like phenomena after chemotherapy treatment are still uncertain (Doll *et al.*, 1986). Contrary to Vogelzang *et al.*'s observation (1981) we did not find any correlation with smoking habits. Four of the 32 patients who received only surgical treatment also reported Raynaud-like phenomena. Three of these patients emphasised that their symptoms were limited to their feet. Thrombangitis obliterans was diagnosed in one of them. In two patients, both of whom had undergone unilateral RLND, supplementary investigations revealed autonomic dysfunction in the contralateral leg.

Five of 31 patients from subgroup 1 (RLND only) reported symptoms of peripheral sensory 'neuropathy'. Surprisingly, the frequency of peripheral sensory 'neuropathy' did not increase when only three to four cycles of chemotherapy were given in addition to surgery (subgroup 3). However, the combination of radiotherapy and chemotherapy seems particularly detrimental with regard to this side-effect.

No effective treatment exists for Raynaud-like phenomena and peripheral neuropathy. Fortunately the symptoms seem to subside spontaneously with time in some patients, while others gradually get accustomed to them. However, in some patients these side-effects remain disabling, forcing individual patients to change their employment. In an attempt to reduce the neurological side-effects vinblastine has been replaced by VP-16 in the chemotherapy given to testicular cancer patients. This has reduced the acute toxicity (Williams *et al.*, 1987) and will, it is hoped, lead to a decrease in long-term side-effects.

Diffuse abdominal symptoms are common, with roughly one-third of the general population reporting minor abdominal symptoms, such as alternating stools, disturbing abdominal rumbling and colic (Hollnagel *et al.*, 1982; Nyrén, 1985). Though our results are not quite comparable to these reports due to different evaluation methods, our overall percentage of 40% reporting gastrointestinal symptoms after therapy does not seem to be particularly high. However, 35% of the patients who had no symptoms before therapy developed gastrointestinal disturbances after treatment.

Moderate to severe degrees of post-irradiation gastrointestinal side-effects are well known from the literature (Roswit *et al.*, 1972; Hanks *et al.*, 1981; Gallez-Marchal *et al.*, 1984; Langlois *et al.*, 1985; Coia *et al.*, 1988), and a dose–response relationship has been shown (Friedman *et al.*, 1952; Coia *et al.*, 1988; Fosså *et al.*, 1989*b*). The increased risk of developing such toxicity is demonstrated in our study by the fact that eight of nine patients who regularly used medications for diverse digestive disorders had been irradiated. Dependent on fractionation schemes, total dose, treatment volume and observation time about 5–9% of irradiated patients will develop moderate to severe post-treatment gastrointestinal disorders (Hamilton *et al.*, 1987; Coia *et al.*, 1988), and in 5–9% peptic ulcer is found (Hamilton *et al.*, 1982; Fosså *et al.*, 1989*b*. The frequency of gastrointestinal toxicity reported from other studies is thus lower than found in the present series in which mild degrees of toxicity are also evaluated. Based on our study, the combination of radiotherapy and cytostatic drugs in particular seems to increase the number of moderate to severe gastrointestinal problems, a finding which is contradictory to other reports.

Infertility is one of the major concerns in testicular cancer patients, especially in the younger non-seminoma patients. The problem is partly due to the germ cell malignancy *per se*, but is also related to the type and intensity of treatment, as demonstrated by comparing subgroup 1 with subgroups 3 and 4. From previous studies it is known that three to four cycles of cisplastin-based chemotherapy, as given in subgroup 3, allow recovery of spermatogenesis (Fosså *et al.*, 1985*a*). It is primarily the extent of retroperitoneal surgery and the frequency of 'dry ejaculation' which reduce the chances for post-treatment paternity. Probably the combination of chemotherapy and radiotherapy also play an important but less significant role. Although not specifically addressed in the present study, we know from previous studies that 80–90% of the patients undergoing bilateral RLND (majority of patients in subgroup 3) have 'dry ejaculation', compared to only 20% in patients operated with unilateral RLND (subgroup 1) (Fosså *et al.*, 1985*b*). It is hoped that recently developed nerve-sparing techniques for RLND will allow more young non-seminoma patients to father children after treatment for metastatic testicular cancer.

Ototoxicity is a well-known side effect after treatment with cisplatin, and its frequency increases with increasing cumulative dose (von Hoff *et al.*, 1979; Loehrer *et al.*, 1984).

However, the present study shows that high frequency hearing loss caused by cisplatin is not noticed by most of the patients. Bleomycin-induced decreased lung function was not a major problem for our patients as observed by others (Ginsberg *et al.*, 1982; van Barneveld *et al.*, 1985). One reason may be that our cumulative bleomycin dose did not exceed 300 mg.

In conclusion, the long-term somatic side-effects 3–7 years (mean 4.6 years) after treatment for testicular cancer were on an acceptable level. Few serious complications were reported. However, 30–50% of the patients developed minor somatic complaints which did not seem to affect their general health. In the future one should avoid treatment with both chemotherapy and radiotherapy as this combination increases the frequency of late toxicity. Furthermore, RLND should be as limited as possible in order to preserve fertility. In general, low risk groups and high risk groups of patients should be identified. Less treatment can probably be given to the former whereas intensive treatment is necessary for the latter.

We are grateful to Brit Moe for help in collecting the clinical data. The study was financially supported by the Norwegian Cancer Society.

**References**

COIA, L.R. & HANKS, G.E. (1988). Complications from large field intermediate dose infradiaphragmatic radiation: an analysis of the patterns of care outcome studies for Hodgkin's disease and seminoma. *Int. J. Radiat. Oncol. Biol. Phys.*, **15**, 29.

DOLL, D.C., RINGENBERG, Q.S. & YARBRO, J.W. (1986). Vascular toxicity associated with antineoplastic agents. *J. Clin. Oncol.*, **4**, 1404.

DUNCAN, W. & MUNRO, A.J. (1987). The management of testicular seminoma: Edinburgh 1970–1981. *Br. J. Cancer*, **55**, 443.

FOSSÅ, S.D., OUS, S., ÅBYHOLM, T., NORMAN, N. & LOEB, M. (1985*a*). Post-treatment fertility in patients with testicular cancer. II. Influence of cis-platin-based combination chemotherapy and of retroperitoneal surgery on hormone and sperm cell production. *Br. J. Urol.*, **57**, 210.

FOSSÅ, S.D., OUS, S., ÅBYHOLM, T. & LOEB, M. (1985*b*). Post-treatment fertility in patients with testicular cancer. I. Influence of retroperitoneal lymph node dissection on ejaculatory potency. *Br. J. Urol.*, **57**, 204.

FOSSÅ, S.D., BORGE, L., AASS, N. & 3 others (1987). The treatment of advanced metastatic seminoma: experience in 55 cases. *J. Clin. Oncol.*, **5**, 1071.

FOSSÅ, S.D., AASS, N. & KAALHUS, O. (1989a). Radiotherapy for testicular seminoma stage I. Treatment, results and long-term post-irradiation morbidity in 365 patients. *Int. J. Radiat. Oncol. Biol. Phys.*, **16**, 83.

FOSSÅ, S.D., AASS, N. & KAALHUS, O. (1989*b*). Long-term morbidity after infradiaphragmatic radiotherapy in young men with testicular cancer. *Cancer* **64**, 404.

FRIEDMAN, M. (1952). Calculated risks of radiation injury of normal tissue in the treatment of cancer of the testis. Proc. 2nd. Natl. Cancer Conf., p. 390.

GALLEZ-MARCHAL, D., FAYOLLE, M., HENRY-AMAR, M., LE BOURGEOIS, J.P., ROUGIER, P. & COSSET, J.M. (1984). Radiation injuries of the gastrointestinal tract in Hodgkin's disease: the role of exploratory laparotomy and fractionation. *Radiother. Oncol.*, **2**, 93.

GINSBERG, S.J. & COMIS, R.L. (1982). The pulmonary toxicity of antineoplastic agents. *Semin. Oncol.*, **9**, 34.

HAMILTON, C.R., HORWICH, A., BLISS, J.M. & PECKHAM, M.J. (1987). Gastro-intestinal morbidity of adjuvant radiotherapy in stage I malignant teratoma of the testis. *Radiother. Oncol.*, **10**, 85.

HANKS, G.E., HERRING, D.F. & KRAMER, S. (1981). Patterns of care out-come studies: results of the national practice in seminoma of the testis. *Int. J. Radiat. Oncol. Biol. Phys.*, **7**, 1413.

HOLLNAGEL, H., NØRRELUND, N. & LARSEN, S. (1982). Occurrence of abdominal symptoms in a 40 year-old population in Glostrup. *Ugeskr. Laeger*, **144**, 267.

KLEPP, O., FOSSÅ, S.D., OUS, S. & 5 others (1984). Multi-modality treatment of advanced malignant germ cell tumours in males. I. Experience with cis-platinum-based combination chemotherapy. *Scand. J. Urol. Nephrol.*, **18**, 13.

LANGLOIS, D., LE BOURGEOIS, J.P., LEUNG, S. & KUENTZ, M. (1985). Intestinal complications of wide field abdominal irradiation for lymphoma. *Radiother. Oncol.*, **3**, 292.

LOEHRER, P.J. & EINHORN, L.H. (1984). Diagnosis and treatment. Drugs five years later. Cisplatin. *Ann. Intern. Med.*, **100**, 704.

NYRÊN, O. (1985). Non-ulcer dyspepsia. PhD thesis. *Acta Universitatis Upsaliensis*, **527**.

PECKHAM, M.J., BARRETT, A., MCELWAIN, T.J. & HENDRY, W.F. (1979). Combined management of malignant teratoma of the testis. *Lancet*, **ii**, 267.

ROSWIT, B., MALSKY, S.J. & REID, C.B. (1972). Severe radiation injuries of the stomach, small intestine, colon and rectum. *Am. J. Roentgenol.*, **114**, 460.

ROTH, B.J., GREIST, A., KUBILIS, P.S., WILLIAMS, S.D. & EINHORN, L.H. (1988). Cisplatin-based combination chemotherapy for disseminated germ cell tumours: long- term follow-up. *J. Clin. Oncol.*, **6**, 1239.

VAN BARNEVELD, P.W.C., MULDER, N.H., VAN DER MARK, T.W. & SLEIJFER, D.T. (1985). Bleomycin and pulmonary toxicity. *Neth. J. Med.*, **28**, 516.

VOGELZANG, N.J., BOSL, G.J., JOHNSON, K & KENNEDY, B.J. (1981). Raynaud's phenomenon: a common toxicity after combination chemotherapy for testicular cancer. *Ann. Intern. Med.*, **95**, 288.

VOGELZANG, N.J., TORKELSON, J.L. & KENNEDY, B.J. (1985). Hypomagnesemia, renal dysfunction, and Raynaud's phenomenon in patients treated with cisplatin, vinblastine and bleomycin. *Cancer*, **56**, 2765.

VON HOFF, D.D., SCHILISKY, R., REICHERT, C.M. & 4 others (1979). Toxic effects of *cis*-dichlorodiammineplatinum (II) in man. *Cancer Treat. Rep.*, **63**, 1527.

WILLIAMS, S.D., BIRCH, R., EINHORN, L.H., IRWIN, L., GRECO, F.A. & LOEHRER, P.J. (1987). Treatment of disseminated germ-cell tumors with cisplatin, bleomycin, and either vinblastine or etoposide. *N. Engl. J. Med.*, **316**, 1435.